# 409. Unvaccinated Adolescents Bear the Majority of Disease Burden of Severe COVID-19 Among Those Admitted to the Intensive Care Unit (ICU)

**DOI:** 10.1093/ofid/ofad500.479

**Published:** 2023-11-27

**Authors:** Ashlesha Kaushik, Corey Thieman, Sandeep Gupta

**Affiliations:** UnityPoint Health and University of Iowa Carver College of Medicine, Sioux City, Iowa; Unity Point Health at St. Luke's Regional Medical Center, Sioux City, Iowa; Pulmonary and Critical Care Medicine, Unity Point Health, Sioux City, Iowa

## Abstract

**Background:**

COVID-19 vaccination is one of the most important public health tools to prevent severe COVID-19.

**Methods:**

COVID-19 vaccine (Pfizer-BioNTech) first received the U.S. Food and Drug Administration (FDA) Emergency use authorization (EUA) for individuals 16 years of age and older on 12/11/2020 and for 12-15 years of age on 5/10/2021. We evaluated clinical and epidemiologic characteristics of critically ill adolescents with COVID-19 admitted to the intensive care unit (ICU) at our tertiary-care centers in Northwestern Iowa (encompassing Sioux City and Sergeant Bluff locations) during the 1-year post-vaccination approval period from June 1, 2021- May 31, 2022. Adolescence was defined as age-group 12-21 years per WHO definition.

**Results:**

Twenty-one adolescents were admitted to the ICU with a median age of 16 years (IQR 12-20 years) and male: female ratio of 1:1.3. The majority (77%) were admitted with COVID-19 related pneumonia and 10 developed hypoxemic respiratory failure; 19 of 21 (90%) adolescent patients admitted to the ICU during this period were unvaccinated and all adolescents developing respiratory failure (10/10, 100%) were unvaccinated. Majority of patients with respiratory failure (70%) required mechanical ventilation (MV). Seventy % of patients with respiratory failure were obese (BMI of >30); 40% of patients had two or more pre-existing comorbidities; those with BMI >40 were 4.3 times more likely to develop respiratory failure (p< 0.0001). Inflammatory markers were 5-20 times higher among patients with respiratory failure (p< 0.05); 60% of those with respiratory failure developed complications including ARDS, acute renal injury and cerebral anoxic encephalopathy. All with respiratory failure required parenteral steroids. Mean duration of ICU stay was significantly longer for patients with respiratory failure [14.6 (±1.7) days versus 7.5 (± 2.6) days, p< 0.0001)]. None of the patients died. After hospital discharge, 30% patients reported post COVID-19 fatigue at 3-month follow up.

**Conclusion:**

Our results showed that all of the critically ill adolescent patients with COVID-19 associated hypoxemic respiratory failure were unvaccinated and majority developed complications. These results further emphasize importance of COVID-19 vaccination efforts for adolescents.

**Disclosures:**

**All Authors**: No reported disclosures

